# Identification of a novel homozygous mutation in the *DDR2* gene from a patient with spondylo-meta-epiphyseal dysplasia by whole exome sequencing

**DOI:** 10.22038/IJBMS.2020.44487.10405

**Published:** 2021-02

**Authors:** Masoud Heidari, Morteza Soleyman-Nejad, Alireza Isazadeh, Mohammad Hossein Taskiri, Manzar Bolhassani, Nahid Sadighi, Zahra Shiri, Zahra Karimi, Mansour Heidari

**Affiliations:** 1 Department of Animal Biology, Faculty of Natural Sciences, University of Tabriz, Tabriz, Iran; 2 Ariagene Medical Genetics Laboratory, Mahmoudnejad Ave, Qom, Iran; 3 Immunology Research Center, Tabriz University of Medical Sciences, Tabriz, Iran; 4 Advanced Diagnostic and Interventional Radiology Research Center (ADIR), Tehran University of Medical Sciences (TUMS), Tehran, Iran; 5 Department of Medical Genetics, Tehran University of Medical Sciences (TUMS), Tehran, Iran

**Keywords:** DDR2 gene, In silico, Sanger sequencing, Splice-site mutation, Spondylo-meta-epiphyseal - dysplasia, Whole exome sequencing

## Abstract

**Objective(s):**

The spondylo-meta-epiphyseal dysplasia (SMED) short limbs-hand type is a rare autosomal recessive disease, which is characterized by premature calcification leading to severe disproportionate short stature and various skeletal changes. Defective function of a conserved region encoding discoidin domain receptor tyrosine kinase 2 (DDR2 protein) by the discoidin domain-containing receptor 2 (*DDR2 *gene) is cause of this disease. The purpose of present study was to investigate disease-causing mutations on *DDR2* gene in an Iranian family with SMED, and predict the DDR2 protein molecular mechanism in development of SMED.

**Materials and Methods:**

In the present study, we evaluated a 2-year-old male with SMED. Detection of genetic changes in the studied patient was performed using Whole-Exome Sequencing (WES). PCR direct sequencing was performed for analysis of co-segregation of variants with the disease in family. Finally,* in silico* study was performed for further identification of molecular function of the identified genetic variant.

**Results:**

We detected a novel splice-site mutation (NM_001014796: exon9: c.855+1G>A; NM_006182: exon8: c.855+1G>A) in *DDR2* gene of the studied patient using WES. This mutation was exclusively detected in patients with homozygous SMED, not in healthy people. The effects of detected mutation on functions of *DDR2* protein was predicted using *in silico *study.

**Conclusion:**

The causative mutation in studied patient with SMED was identified using Next-generation sequencing (NGS), successfully. The identified novel mutation in *DDR2* gene can be useful in prenatal diagnosis (PND) of SMED, preimplantation genetic diagnosis (PGD), and genetic counseling.

## Introduction

The spondylo-meta-epiphyseal dysplasia (SMED) short limbs-hand type is an uncommon autosomal recessive disease with various changes in skeletal growth. This disease is characterized by platyspondyly, disproportionately short stature, shortening of the lower and upper limbs, abnormal epiphyses and metaphyses, punctate calcifications, and short broad fingers (1, 2). The bone dysplasia in SMED patients is progressive with severe complications, which lead to death in some patients. The atlantoaxial instability resulting in cord damage was the most important cause of death in patients with SMED (2, 3). 

In previous studies, various homozygous mutations have been identified on the discoidin domain-containing receptor 2 (*DDR2* gene) as the main cause of severely dwarfing condition in patients with SMED (4, 5). The *DDR2* gene encodes a receptor tyrosine kinase (RTK), known as discoidin domain receptor tyrosine kinase 2 (DDR2 protein), which identify and interact with collagen as their ligands (6). After collagen binding, the receptor displays delayed and sustained tyrosine phosphorylation which further elicits downstream signaling to cellular metabolic pathways that cross-talk at various points (7, 8). Also, dysregulation of *DDR2 *gene was associated with various diseases, such as cancer, arthritis, and fibrosis. On the other hands, *DDR2* gene developed as potential therapeutic targets (9).

The DDR2 protein is produced by mesenchymal cells and is activated by collagen X and fibrillar collagens (6, 10). Also, DDR2 protein plays an important role in collagen fibrillogenesis collagen remodeling, regulation of matrix metalloproteases, and invasion (7, 11). A study on DDR2 protein knockout in mice demonstrated the impacts of DDR2 protein in skeletal growth and abnormalities which reflect the phenotype of SMED in humans (12).

The DDR2 protein are identified as plasma membrane RTKs, which consist of a C terminal catalytic tyrosine kinase domain, a large cytosolic juxtamembrane domain, a transmembrane domain (TM), and an extracellular domain (ECD). ECD is necessary for ligand binding and comprising a unique region to DDR2 protein with a DS-like domain and an N-terminal discoidin homology (DS) domain (13). The DDR2 protein are pre-formed homodimers on the membrane of cells, even in the absence of collagen (14). In recent years, significant development and progress has been obtained in understanding and recognition of the collagen structure; although the exact binding partners and signaling pathways in control of bone growth by DDR2 protein remain unknown (15).

So far, there is no study on SMED-cause mutations of *DDR2* gene in the Iranian population. Therefore, in the present study, whole exome sequencing (WES) and sequencing-PCR were applied to the fast and accurate diagnosis of *DDR2* gene mutations in an Iranian patient with SMED and a new pathogenic mutation was found.

## Materials and Methods

***Case presentation and sample collection***

In the present study, a 2 years old Iranian male diagnosed with SMED, referred to Aria Gene Medical Genetics Laboratory (Qom-Iran), was investigated in February 2019. In his family, the proband was offspring of consanguineous marriages. Physical examination showed several problems such as retrognathia and a high-palate, deep eyes with hypotelorism, sparse eyebrows, a long philtrum, a wide nasal bridge with flat nasal tip. Our proband showed a narrow chest, a short neck, and short limbs with short puffy fingers. The nervous system was normal. The skeletal examination showed, a narrow thorax with short broad ribs, platyspondyly with pear-shaped vertebrae, short and broad tubular bones with irregularities at the metaphysis and epiphyses, and short and broad pelvic bones. Hands and feet were short with broad metacarpals, metatarsals, and phalanges. Moreover, 100 healthy controls were selected (as healthy controls), which referred to clinical centers and hospitals affiliated to Tehran University of Medical Sciences for routine examination. To exclude epidemiological bias, the healthy age and ethnically matched as well as unrelated genetically controls were selected from the population of Tehran, Iran. According to the Declaration of Helsinki ethical standards, the studied patient and his parents, as well as healthy controls were informed about the study. The present study was performed with the approval of the Institutional Review Board (IRB) and an informed consent was received from all subjects. The pedigree of the patient was drawn (Cyrillic 2.1 software) to determine the inheritance pattern of the disease.

***Genomic DNA extraction and whole-exome sequencing (WES)***

Peripheral blood sample (5 ml) was received from all subjects, and genomic DNA was extracted using a DNA purification kit (Roche, Switzerland) according to the manufacturer’s instructions. The quantity and quality of the extracted genomic DNA were evaluated using a nanodrop instrument (Thermo Fisher Scientific, USA) and electrophoresis on 1% agarose gel, respectively. The Whole Exome Sequencing (WES) was used to enrich the genomic coding regions, and some other important genomic regions. Next-generation sequencing (NGS) was performed to sequence close to 100 million reads on an Illumina Sequencer (Illumina, San Diego, CA, USA). The bioinformatics analysis of the obtained sequencing results was conducted using the international databases. The genetic variants such as indels and point mutations were identified using SAMtools and analyzed by ANNOVAR software. A candidate gene was considered a variant that fulfilled the following criteria: (i) missense, nonsense, frameshift, and splice site variants, (ii) absent or rare (frequency below 1%) in the two databases (dbSNP, 1000 G), and (iii) homozygous variants in the patient (16). 

***Confirmation and validation of WES results by sanger sequencing***

The target exons containing *DDR2* gene mutations were amplified using polymerase chain reaction (PCR) in 25 μl total volume: 1 μl primer (10 pmole), Taq DNA polymerase (0.2 U), dNTPs (200 μM), 0.67 μl MgCl2 (50 mM), genomic DNA (60 ng), and PCR buffer (2.5 μl). The thermal cycler condition was as follows: 1 cycle initial denaturation (3 min at 95 °C), 35 cycles denaturation (30 sec at 95 °C), 35 cycles annealing (30 sec at 60 °C) 35 cycles extension (30 sec at 72 °C), and 1 cycle final extension (10 min at 72 °C). The PCR products were separated on 2% agarose gels. They were sequenced on an ABI 3130 automated sequencer (Applied Bio systems, Forster City, CA, USA). Sequence data searches were performed in non-redundant nucleic and protein databases BLAST (http://www.ncbi.nlm.nih.gov/BLAST) (17).

***In silico analyses of DDR2 gene mutation***

To investigate whether the identified mutations could affect the biological function of the altered protein, we used two different computational analysis tools. These algorithmic programs included: Sorting Intolerant from Tolerant (SIFT) (https://sift.bii.a-star.edu.sg/www/SIFT_seq_submit2.html) and PolyPhen-2-Polymorphism Phenotyping v2 (http://genetics.bwh.harvard.edu/pph2/)(18).

## Results

***DDR2 gene mutation detection using WES***

WES was performed in the studied SMED patient. In the present study, a novel homozygous splice-site mutation (NM_001014796: exon9: c.855+1G>A; NM_006182: exon8: c.855+1G>A) was detected. 

***Confirmation and validation of WES results using sanger sequencing***

**Figure 1:**
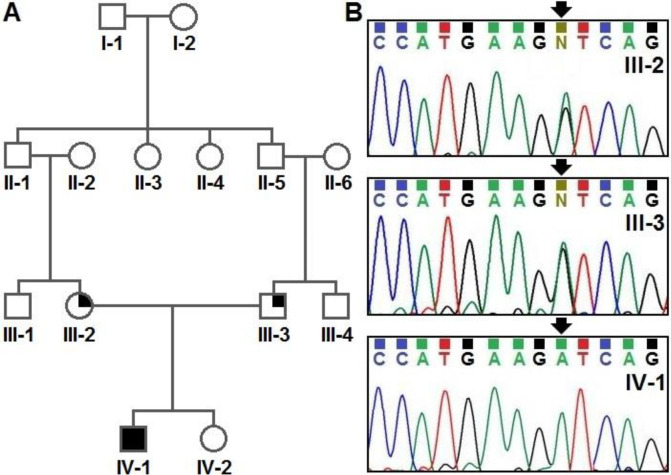
Pedigree analysis and molecular evaluation of a patient with *DDR2* gene mutation. A: The pedigree of the affected patient (arrow indicates the proband). The affected member (IV-1) is homozygous for the mutation and both parents are heterozygous

In order to confirm and validate the novel mutation of *DDR2* gene (NM_001014796: exon9: c.855+1G>A; NM_006182: exon8: c.855+1G>A) detected using the WES method in the studied patient, his family’s members were evaluated using PCR direct sequencing. We found the genetic variants of *DDR2 *gene in the homozygous state in the studied patient, while his parents were heterozygous (Figure 1).

***In-silico study***

We subjected the identified variants in the *DDR2 *gene to two different bioinformatics tools including SIFT and PolyPhen-2 to investigate whether these variants have any biological effects on the DDR2 protein. Based on SIFT findings, genetic variants scoring tolerance index (TI) of ≤0.05 are considered intolerant. PolyPhen-2 results predicted can be classified into three categories, probably damaging, possibly damaging, and benign genetic changes. These predictions are based on position-specific independent count score difference, where score 1 is considered the most damaging. Moreover, to assess the splice site mutation, we utilized the NNSplice software (www.fruitfly.org/seq_tools/splice.html), which showed the score of the donor-site as about 0.86. According to this program, the score of the donor-site is between 0–1, in the presence of splice-site mutation the score gets larger close to 1.

## Discussion

SMED is recognized as an autosomal recessive disorder, which is due to homozygous mutations of *DDR2* gene. This gene encodes RTKs in the plasma membrane, which play a critical role in bone growth (4). Until now, 22 patients have been reported with mutation of *DDR2* gene, but only 7 types of mutations (one splice-site, two deletions, and four missense) have been reported on the conserved region of *DDR2 *gene, which encodes the tyrosine kinase domain. The mentioned mutations on DDR2 gene causing loss of function by at least two mechanisms include targeting plasma membrane and its ligand-binding activity and defects in DDR2 protein (5, 19). 

**Figure 2:**
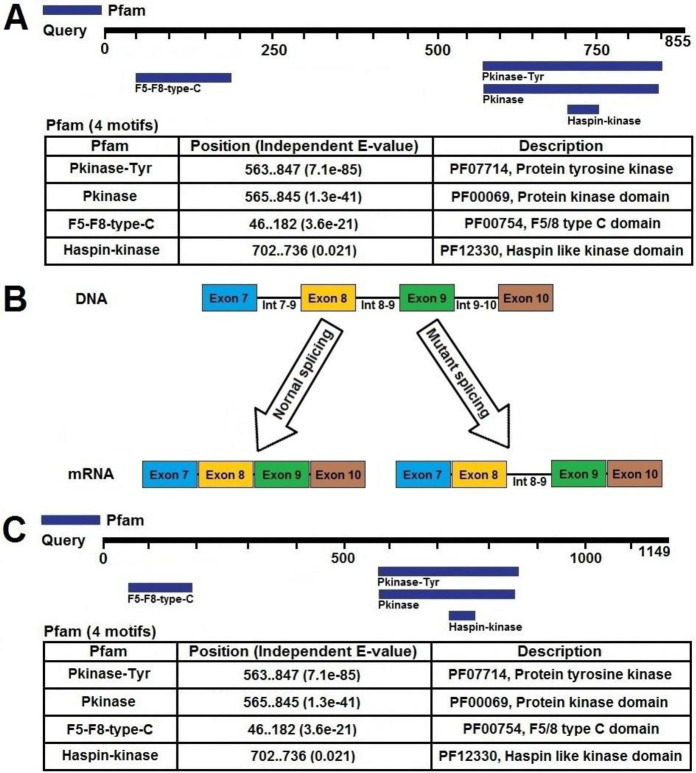
The mutant and normal alternative splicing process and amino acid sequence of *DDR2 *gene. In the normal form, the number of amino acids is 855 (A), but in the mutants due to mutation, the number of amino acids increased by 1,149 (C). A splice donor is a GT pair at the 5' end of an intron that is part of a consensus sequence, and that a splice acceptor is an AG base pair at the 3' end of an intron that is part of a consensus sequence. In the example shown here, the splice donor site mutates via a single base substitution such that it can no longer be a splice donor (B)

In the present study, we used WES to identify a new splice-site mutation (NM_001014796: exon9: c.855+1G>A; NM_006182: exon8: c.855+1G>A) in *DDR2 *gene of a 2 year old Iranian male with clinical symptoms of SMED. The new splice-site mutation (G>A) cause to remains the 8-9 intron in alternative splicing process, which lead to produce a raised protein (the normal protein with 855 amino acids increases to a mutated protein with 1149 amino acids) (Figure 2). Also, the *in silico* bioinformatics analysis (according to evolutionary conservation, nature, and position of amino acid change) of identified mutation, showed that these mutations are able to damage the structure and function of the DDR2 protein. 

The disease-causing mutation on *DDR2* gene leads to residues of amino acids in the tyrosine kinase domain. All of the kinases showed a very similar structure. Most of these kinases contain C-terminal tail; whereas catalytic kinase domains of the DDR2 protein are followed by very short C-terminal tails. The kinase domain structure of the DDR2 protein showed that C-terminal amino acids, assigned to a C-terminal tail, are section of a DDR2 protein α-helix domain, which interacts with other domains (20). Therefore, mutations in tightly folded C-terminus are likely to cause misfolding of the kinase domain and subsequent retention in the rough endoplasmic reticulum. Moreover, the high DDR2 protein molecular weight form has been shown to be inherently unstable and largely dependent on glycosylation for efficient cell membrane trafficking (21).

The two mechanisms lead to loss of DDR2 protein function with mutations occurring, which include mutant proteins retention in the endoplasmic reticulum (ER) and activity loss of ligand binding (5). ER showed an exact system of quality control, which allows only mature proteins with native conformation trafficking. Therefore, misfolded proteins fail to conform with quality control of ER, are enclosed in the ER and then degraded by ubiquitin/proteasomal machinery, which are implicated in the pathogenicity of congenital disorders (22).

The clinical diagnosis of SMED is according to long philtrum, short neck, dysmorphic face, flat nasal tip, wide nasal bridge, and disproportionate short stature (23, 24). The soft tissue and chondral calcifications are often associated with specific radiological signs. This disorder is associated with narrow chest and short ribs, which are the cause of respiratory failure and responsible for death in patients with SMED. The early detection of this complication is required for timely surgical decompression (25). 

DDR2: Discoidin Domain Receptors 2

DDR2: Discoidin Domain Receptors 2

## Conclusion

Our study showed more arguments for WES, the cost-effective and powerful tool for the molecular diagnosis of clinically heterogeneous disorders like SMED. We conclude that the reported genetic variants in *DDR2 *gene strongly provide support for the previous studies suggesting that this gene contains different hotspot regions. However, further studies are required to clarify genotype-phenotype association in patients with deleterious mutations in the *DDR2* gene.
